# Prenatal diagnosis of recurrent moderate skeletal dysplasias in lamin B receptors

**DOI:** 10.3389/fgene.2022.1020475

**Published:** 2023-01-13

**Authors:** Xueping Shen, Zhi Li, Xuekui Pan, Juan Yao, Guosong Shen, Su Zhang, Minyue Dong, Lihong Fan

**Affiliations:** ^1^ Center of Prenatal Diagnosis, Huzhou Maternity & Child Healthcare Hospital, Huzhou, China; ^2^ Women’s Hospital, School of Medicine Zhejiang University, Hangzhou, China; ^3^ Key Laboratory of Reproductive Genetics (Zhejiang University), Ministry of Education, Hangzhou, China

**Keywords:** prenatal moderate skeletal dysplasia, lamin B receptor, NM_002296.4:c.1757G>A, NP_002287.2:p.Arg586His, whole-exome sequencing, genetic counseling

## Abstract

The lamin B receptor (*LBR*) gene is located in chromosome 1q42.12 and encodes the lamin B receptor, an intracellular protein that binds to lamin B. *LBR* mutations are associated with a broad phenotypic spectrum ranging from non-lethal to lethal skeletal dysplasias. The typical phenotypes include the Pelger−Huet anomaly (PHA) and embryonic lethal Greenberg dysplasia (GRBGD). With the further study of this gene, other phenotypes have been found in different individuals. This retrospective study analyzed recurrent prenatal moderate skeletal dysplasias in Chinese fetuses. Nothing malformed was detected in the fetal karyotype and microarray, while the whole-exome sequencing identified a homozygous variant (NM_002296.4:c.1757G>A, NP_002287.2:p.Arg586His) in exon 14 of the *LBR* gene in both fetuses. Mutation analysis in the parents confirmed that the c.1757G>A variation is heterozygous by Sanger sequencing. Intensive analysis on bioinformatics and familial co-segregation suggest that the homozygous variation in the *LBR* gene is responsible for this recurrent prenatal moderate skeletal dysplasia. Moreover, moderate skeletal dysplasias differ from typical GRBGD phenotypes. Our findings are based on the DNA base test and the prenatal diagnosis of skeletal dysplasia, which can be helpful in proper phenotyping and contribute to a better understanding of the correlation between the phenotype and genotype.

## Introduction

The *LBR* is a bifunctional protein encoded by the gene of the *LBR*. It has the dual function of affecting the nucleus division of neutrophils and sterol reductase activity. Mutations in this gene can affect a range of phenotypes. Variants may affect sterol reductase activity, resulting in the failure of cholesterol synthesis and elevated levels of the sterol metabolite cholesta-8, 14-dien-3-ol (accumulated in GRBGD fetuses), resulting in specific skeletal abnormalities in the developing fetus and fatal perinatal dysplasia. It can also cause defects in neutrophil differentiation characterized by a reduced number of nuclear fragments (dumbbell-shaped double-pore nuclei) and coarse aggregation of the nuclear chromatin, resulting in the Pelger−Huet anomaly (PHA) and Reynolds syndrome. There is also a mild skeletal dysplasia, the Pelger−Huet anomaly with a mild skeletal dysplasia (PHASK). The clinical features of 11 fetuses with the Greenberg dysplasia have been reported in the relevant studies, of which biallelic pathogenic variants have been identified in the *LBR* in seven cases (Greenberg et al., 1988; Chitayat et al., 1993; Horn et al., 2000; Waterham et al., 2003; Konstantinidou et al., 2008; Clayton et al., 2010; Giorgio et al., 2019). The clinical manifestations of the other four cases were similar to those of the Greenberg dysplasia, which were caused by the deficiency of the sterol metabolism (Gregersen et al., 2020). Dappled diaphyseal dysplasia in the two fetuses were described in Carty’s report (Carty et al., 1989), Astley–Kendall dysplasia in another two fetuses were described in Astley and Kendall’s report (Astley & Kendall, 1980), and three intermediate phenotype fetuses were described in Elçioglu’s study (Elçioglu and Hall, 1998). Thus, the Greenberg dysplasia has multiple characteristics of a skeletal dysplasia, and its severity varies.

In this study, two fetuses with moderate skeletal dysplasia with homozygous variation c.1757G>A of the *LBR* gene were studied to expand the phenotypic spectrum and further guide the prenatal diagnosis of this disease.

## Patients and methods

### Case presentation

**FIGURE 1:**
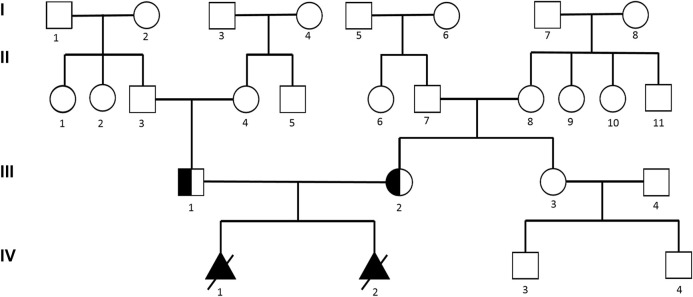
Pedigree of the fetuses.

**FIGURE 2:**
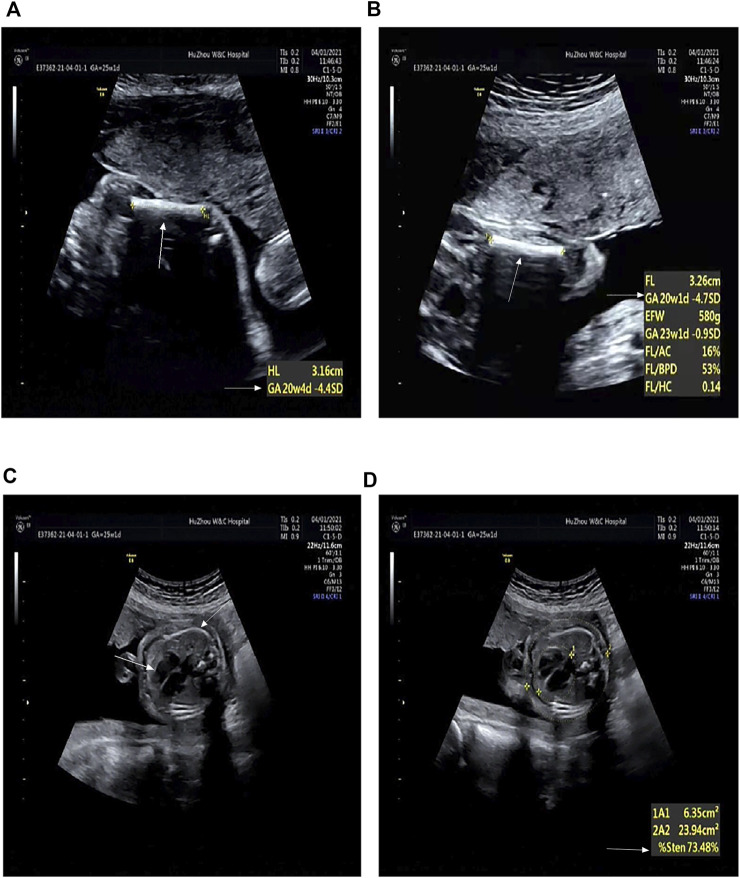
Radiographic features of the first fetus (at 25+1 weeks’ gestation). **(A)** Humerus is smaller by 4.4 SD (arrows). **(B)** Femur is smaller by 4.7 SD (arrows). **(C)** Thoracic area is significantly reduced (arrows). **(D)** Enlarged cardiothoracic ratio (arrows).

**FIGURE 3:**
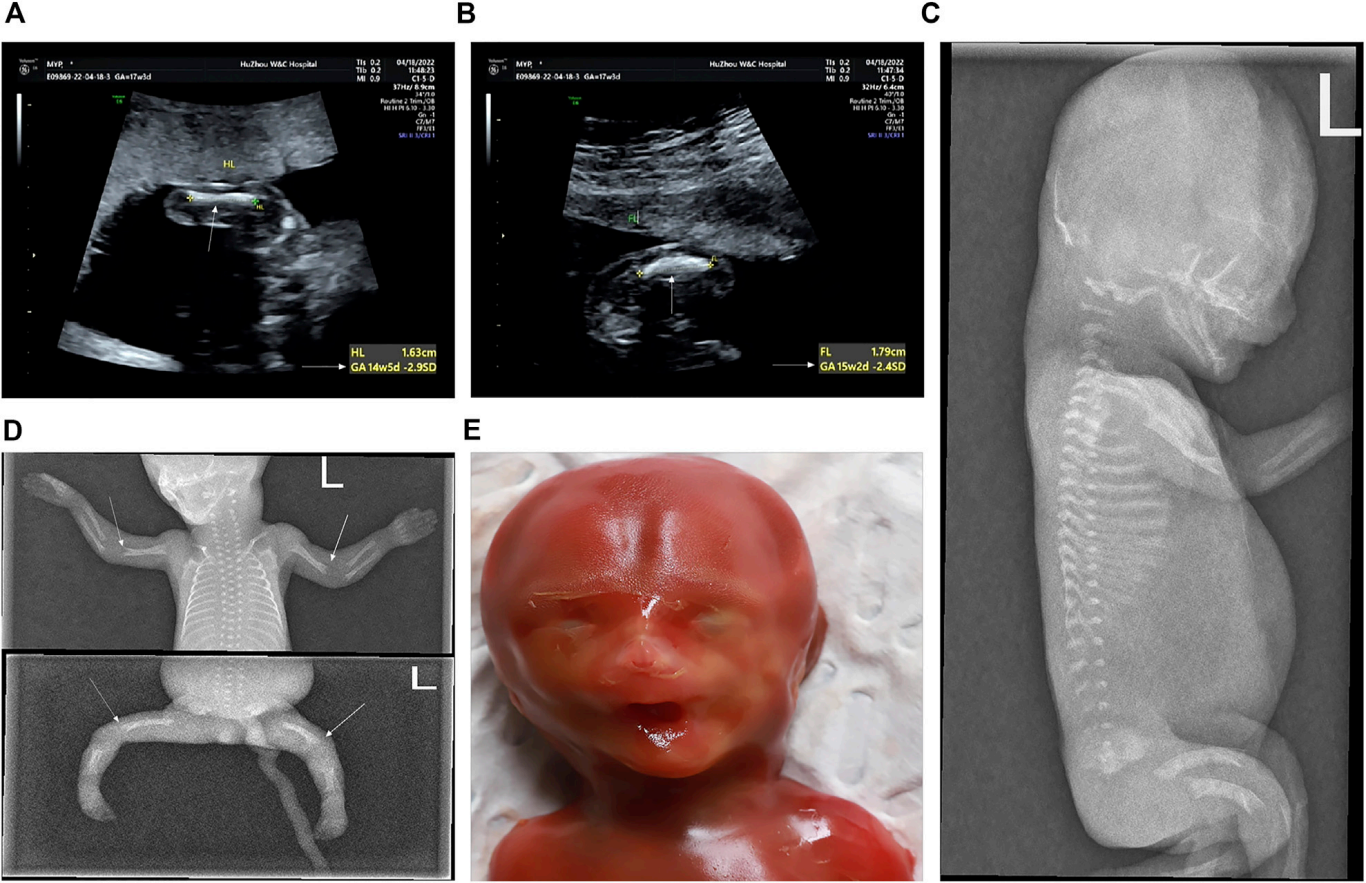
Prenatal and postnatal images of the second fetus (at 17+3 weeks’ gestation). **(A)** Humerus is smaller by 2.9 SD (arrows). **(B)** Femur is smaller by 2.4 SD (arrows). **(C−E)** Features of the second fetus after induction. **(C)** Lateral view of the spine shows flattening of each vertebral body and dysplasia of the epiphysis. **(D)** Elbow joint of the long bone end was misaligned. The multiple densities at the end of the long bones of the extremities were not uniform, and the edge was irregular. Mild moth-eaten skeletal dysplasia is seen in the distal humerus and femur of both sides (arrows). **(E)** In the mugshot of the fetus after induced labor, there was no obvious special face.

A 25-year-old primiparous woman (Ⅲ2, Figure 1) was referred to the prenatal diagnosis center of our researching hospital due to an abnormal ultrasound at 25+1 weeks of gestation. Fetal system ultrasounds suggested that the limb long bones of the fetal are less than 2−4 standard deviations, while the thoracic cage is smaller than normal (Ⅳ1, Figure 2). After the termination of the first pregnancy, the woman had another pregnancy again after 1 year. The second fetus (Ⅳ2) was diagnosed as lethal skeletal dysplasia by prenatal ultrasound and only mild moth-eaten skeletal dysplasia by X-ray after induction at 17+3 weeks of gestation (Figure 3). Hydrops was not detected in both fetuses under ultrasound after labor induction. The patient did not have an MRI and refused to perform an autopsy on the fetus. There was no nuclear lobulation in the peripheral blood of the parents, only leaving the fetus unexamined. The woman denied any exposure to teratogenic chemicals, radiation, nicotine, or alcohol before or during the pregnancy. The relevance of the family congenital inheritance was also denied. The research was approved by the Ethics Committee of Huzhou Maternity & Child Health Care Hospital. Informed consent brief was signed by the parents for the examination of the fetus, as was done by all the other participants.

### Acquisition of the amniotic fluid

Within the attendance, projected amniotic fluid was obtained through an ultrasound-guided puncture under sterile conditions at 25+1 and 17+3 weeks of gestation, respectively. A volume of 30 ml amniotic fluid was collected, of which 15 ml was used for cell culture chromosome karyotype preparation, and the other 15 ml was used for DNA extraction for subsequent experiments.

### Karyotype analysis

Fetal exfoliated epithelial cells in the amniotic fluid were prepared in an appropriate medium (BIO-AMF^TM^-2) and afterward cultured at 37°C with the condition of 5% CO_2_ for a week. Soon after subculturing, G-banding was performed at the 400-band level through treatment with 0.1 mg/ml colchicine and hypotonic (trisodium citrate 10%) fixation (glacial acetic acid: methanol = 1:3), with the analysis under the conditions of a microscope. On the basis of the International System for Human Cytogenomic Nomenclature (2016), 20 chromosomes were dated, meanwhile five karyotypes were analyzed as well.

### Chromosomal microarray analysis

A total of 250 mg fetal DNA was extracted from the amniotic fluid, and analyzed by the CytoScan™ HD whole-genome SNP array (Affymetrix, United States), including 750,000 SNP probes and 1,950,000 CNV probes. The experiment contains quality control, enzyme digestion, PCR, purification, labeling, hybridization, and scanning. All the data were analyzed by the Chromosome Analysis Suite 4.2 based on the conditions of the instructions of the manufacturers.

### Whole-exome sequencing and Sanger sequencing

Genomic DNA was extracted from the amniotic fluid of the two fetuses and peripheral blood of the parents, which was used for the whole-exome sequencing. Exome capture was performed using the SureSelect Human All Exon V4 kit (Agilent Technologies, Santa Clara, CA, United States), followed by sequencing using the Illumina NovaSeq 6000 system (Illumina, San Diego, CA, United States). After sequencing and filtering out the low-quality reads, the high-quality reads were compared to the GRCh37/hg19 reference human genome using Sentieon BWA (Sentieon, United States) with the MEM align method, and the only variants located in the coding sequence as the only splice site regions were retained. Variant calling was performed using the Genome Analysis Tool Kit (GATK v4.0). Afterward, the candidate variants, including single-nucleotide variants (SNVs) and indels, were filtered by frequencies of specific databases, including the Human Gene Mutation Database (HGMD), ClinVar database, 1000 Genomes Project, Exome Aggregation Consortium (ExAC), Exome Sequencing Project 6500 (ESP6500), database of single-nucleotide polymorphisms (dbSNP), and Genome Aggregation Database (gnomAD). Mutation sites, known as polymorphic sites, were excluded, and the variants with allele frequency ≤1% were retained. Here, it can be predicted that the effect of variants is affected by SIFT, MutationTaster, and PROVEAN. The 3D-folded structure of the encoded protein was predicted, and the effects of mutations were predicted by AlphaFold (https://alphafold.ebi.ac.uk/). The interpretation of sequence variants was performed according to the American College of Medical Genetics and Genomics (ACMG). The online Clustal Omega (https://www.ebi.ac.uk/Tools/msa/clustalo/) was used to analyze the evolutionarily conserved sequences among species (human, gorilla, dog, mouse, elephant, armadillo, chicken, X-tropicalis and zebrafish). Ultimately, the variants were confirmed through Sanger sequencing and amplification of potentially mutated sequences. The primer sequences (Table 1) were designed by Primer3 (http://primer3.ut.ee/). PCR products were sequenced using the ABI 3500DX and further analyzed using DNASTAR 5.0 software (Fan et al., 2022).

**TABLE 1:** Primer sequences used to simplify *LBR* genomic fragments.

Name of the primer	Primer sequences (5′−3′)
Exon 14-*LBR*-F	ACC​TTC​TGC​CAC​TTT​CCA​GT
Exon 14-*LBR*-R	CAG​CTG​GAA​TTG​CAG​GAG​TA

## Results

### Identification and bioinformatics prediction of c.1757G>A in the *LBR*

**FIGURE 4:**
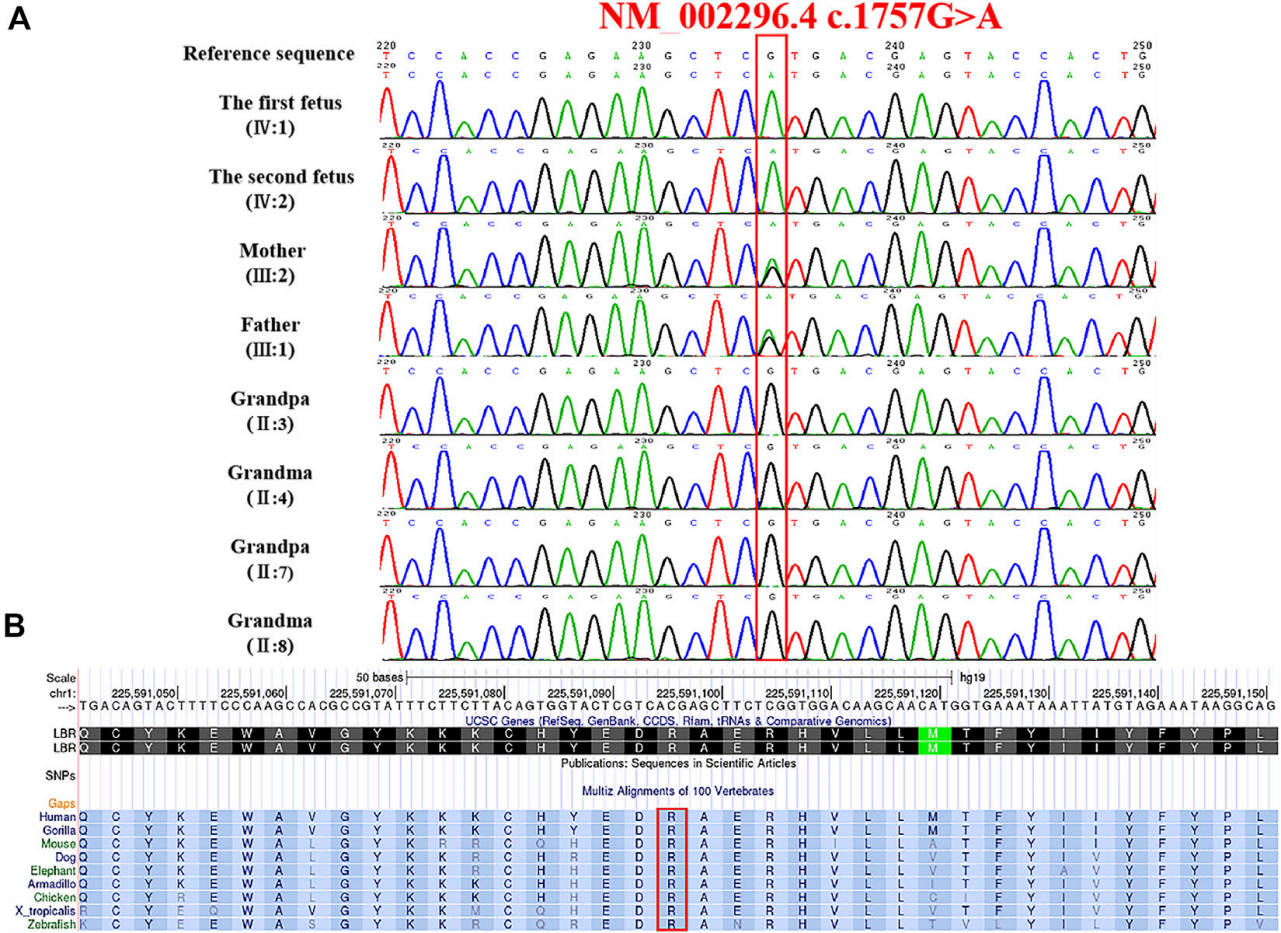
Genetic variation identified in these cases. **(A)** Variation detected in two fetuses and parents. **(B)** Conservation of the mutant amino acid (Pro586) in different species (human, gorilla, dog, mouse, elephant, armadillo, chicken, X-tropicalis, and zebrafish).

Karyotype and CMA analysis showed no abnormalities. Subsequently, our group performed the whole-exome sequence to detect the presence of any mutations. Nearly 20,000 genes in the human genome were detected using the capture high-throughput chip technology for sequencing. The results showed that a homozygous mutant in the *LBR* (chr1:225591096, NM_002296.4:c.1757G>A, NP_002287.2:p.Arg586His) in both fetuses, which were inherited from their parents; a missense mutation caused by the nucleotide G at position 1757 of the gene coding sequence was changed to A, which led to the arginine at position 586 to be changed to histidine. Sanger sequencing confirmed the variant and showed that the *LBR* variant in this family was consistent with the co-segregation of this disease (Figure 4A).

**FIGURE 5:**
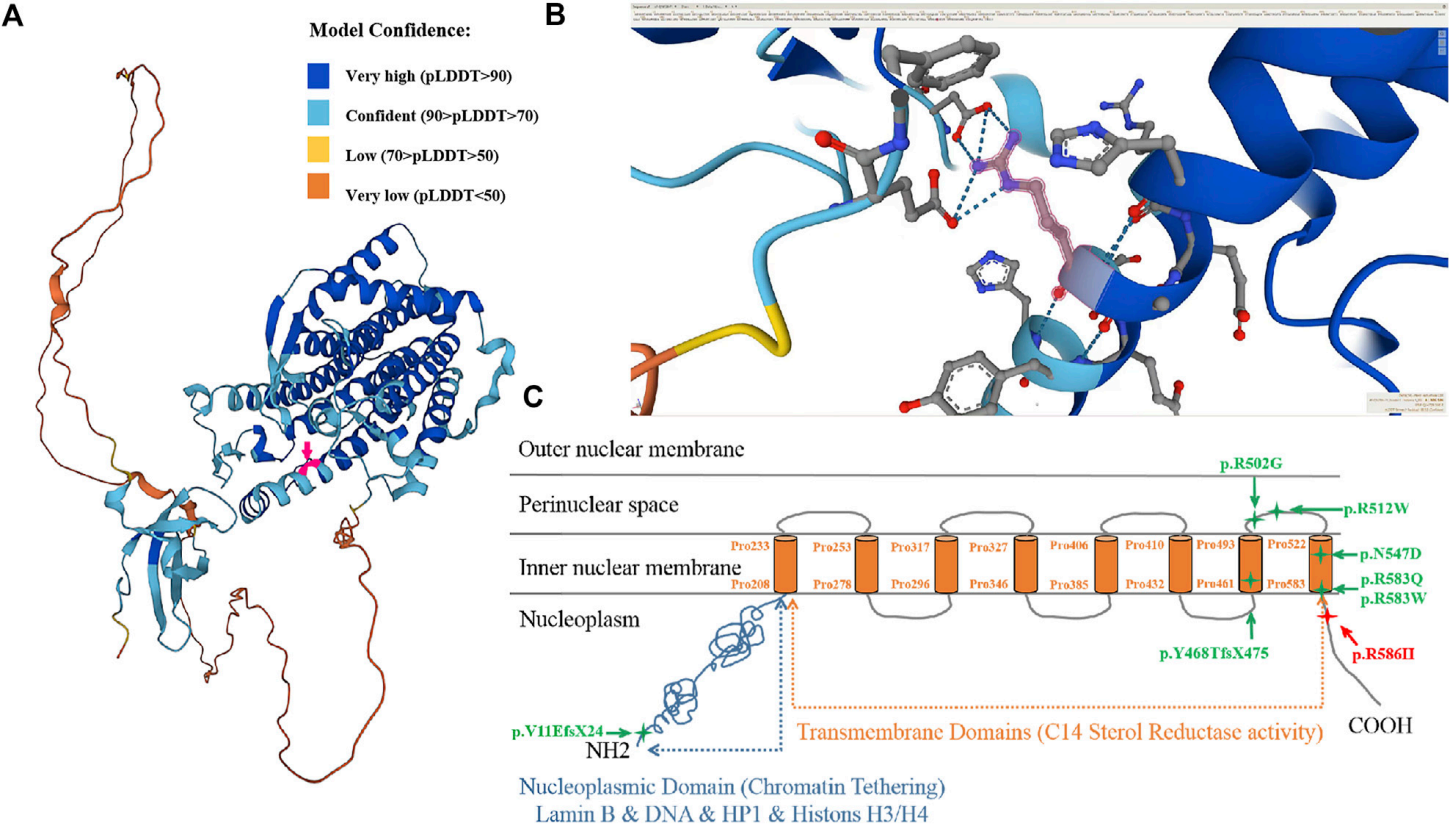
3D protein simulation of 3-beta-hydroxysterol delta 14-reductase encoded by the *LBR* in AlphaFold. **(A)** Overall structure of 3D protein simulation and the localization of the mutated amino acid (red arrow, Pro586 is confident) (model confidence: dark blue is very high, pLDDT>90; light blue is confident, 90 > pLDDT>70; yellow is low, 70 > pLDDT>50; orange is very low, pLDDT<50). **(B)** Enlarged view of the molecular structure of the mutant amino acid (Pro586, red arrow). **(C)** Schematic view of the lamin B receptor as a protein in the nuclear envelope and the location of identified mutations. The missense mutation p.R586H is located near the 8th transmembrane domain (sterol reductase domain).

The frequency of the mutation in both the general population and the East Asian population is less than 1% in GnomAD and ExAC databases. The 1000 Genomes database showed that the frequency of the variation in the general population is < 1% and in the East Asian population is 1%. Both fetuses with a dysplasia of the bone carrying a heterozygous variant c. 1757G>A were reported in this study. This mutation is recorded in the HGMD database (CM136799). In the ClinVar database, only one record showed that the mutation is pathogenic, and the other one is of uncertain significance. It has been reported that one heterozygous variant c.1757G>A in the *LBR* gene was detected in a patient with bilobed neutrophil nuclei and a mild skeletal dysplasia phenotype; another heterozygous variation in the *LBR* gene was detected in the trans position (Borovik et al., 2013). The amino acid sequence alignment of multiple species confirmed that the mutation was 100% conserved (Figure 4B). According to AlphaFold, we confirmed that the Arg586 is located at a conserved site in the 3D folding of 3-β-hydroxysterol δ14-reductase and found that Arg586 is located at one of α-helix (Figure 5A). It also combines with ASP450 and GLU208 as a donor of hydrogen bonds (Figure 5B) (pLDDT score: 88.50). However, we have not scored this mutation through the DeepMind pipeline because there are several limitations in the current implementation of AlphScore. The functional prediction results of the variants by the SIFT, PROVEAN, and MutationTaster tools are shown in Table 2. According to the American College of Medical Genetics and Genomics (ACMG) guidelines for the interpretation of sequence variants, we considered this variant to be likely pathogenic (PM2_Supporting + PP3_Moderate + PM3_Strong + PP4).

**TABLE 2:** *In silico* analysis of the *LBR* variant c.1757G>A.

Variant	MutationTaster		PROVEAN		SIFT	
	Score	Prediction	Score	Prediction	Score	Prediction
c.1757G>A	1	Disease causing	−4.44	Damaging	0.001	Damaging

### Prenatal diagnosis and genetic counseling

Based on the effect of *LBR* mutations and co-segregation analysis of this disease, it was speculated that the homozygous mutation might be responsible for the cause of prenatal lethal bone dysplasia in this Chinese family. Eventually, the couple made the decision to terminate the pregnancy after deep consideration and planned to use pre-implantation prenatal diagnosis to have a healthy baby.

## Discussion

The *LBR* gene was first reported by Schulerin in 1994. It is 35 kb in size and encodes a lamin B receptor with 615 amino acid residues. The *LBR* is a nuclear membrane protein that functions as a 3-beta-hydroxysterol delta 14-reductase and belongs to the ERG4/ERG24 family.

Exons 2−5 encode N-terminal nucleoplasmic domains that interact directly or indirectly with the chromatin, lamina, heterochromatin proteins, histones, and other nuclear components to maintain the structural integrity of lamina networks and immobilize the heterochromatin within the nuclear membrane. Pathogenic aberrance with PHA is located in nucleoplasmic domains or whole genes. Exons 6–14 encode eight C-terminal hydrophobic transmembrane domains, belonging to the sterol reductase family, which participates in cholesterol synthesis through sterol C14-reductase activity and realizes the metabolic function of the *LBR*. Pathogenic variants in the transmembrane domain of the *LBR* may affect sterol reductase activity, leading to a failure of cholesterol synthesis that increase and accumulate the sterol metabolite cholesta-8, 14-dien-3-ol *in vivo*, leading to specific skeletal abnormalities in the developing fetus and a perinatal lethal skeletal dysplasia (Borovik et al., 2013). It has been reported that the aberrance of the pathogen in some cases of skeletal dysplasia are located in hydrophobic transmembrane regions (Nikolakaki et al., 2017). Up to now, there are 38 disease-associated *LBR* gene variants in the database, but only five cases of GRBGD have been reported (Table 3; Figure 5C).

**TABLE 3:** Pathogenic variation of the *LBR* gene in the five reported cases of GRBGD.

Reports	Case	Variation information	Functional changes	Clinical features
			Zygote type	
Peter Clayton et al.	Fetus A	c.1402delT:p.Tyr468Thrfs*475	Nonsense mutation	1. Narrow thorax 2. Generalized hydrops fetalis 3. Ectopic calcification centers4. Intrauterine growth retardation 4. Intrauterine growth retardation 5. Long bones were severely shortened and moth-eaten bones
			Homozygous	
	Fetus B	c.32_35delTGGT:p.Val11Glufs*24	Nonsense mutation	
			Heterozygous	
		c.1748G>A:p.Arg583Gln	Missense mutation	
			Heterozygous	
	Fetus C	c.1639A>G:p.Asn547Asp	Missense mutation	
			Homozygous	
Eliza Thompson et al.	Patient 1	c.1504C>G:p.Arg502Gly	Missense mutation	1. Short stature2. Dysplasia of the metaphysis of the spine
			Heterozygous	
		c.1748G>T:p.Arg583Leu	Missense mutation	
			Heterozygous	
	Patient 2	c.1534C>T:p.Arg512Trp	Missense mutation	
			Homozygous	

Clayton et al., 2010 collected three GRBGD fetuses with the identical clinical characteristics, all of which presented with thoracic spinal stenosis, generalized hydrops fetalis, ectopic calcification center, intrauterine growth retardation, severely shortened long bones and moth-eaten bones, and carrying nonsense or missense variants in the *LBR* gene (Clayton et al., 2010). Eliza Thompson *et al.* reported that two patients with a moderate severity of skeletal dysplasia and spontaneous continuous improvement for the first time, with clinical phenotypes of short stature and spinal metaphyseal dysplasia (Thompson et al., 2019). It can be concluded that GRBGD caused by *LBR* gene variants has a wide range of phenotypic heterogeneity, especially skeletal abnormalities. The reports about exon 14 of the *LBR* gene in the literature are very rare, including only five cases of defects (Best et al., 2003; Mchugh et al., 2011; Clayton et al., 2010; Borovik et al., 2013; Thompson et al., 2019) (Table 4). The clinical manifestations range from isolated PHA to mild skeletal dysplasia PHA and then to the Greenberg skeletal dysplasia.

**TABLE 4:** Reported cases of the *LBR* gene exon 14 mutation.

Case	Reports	Variation information	Functional changes	Disease	Clinical features
			Zygote type		
#1	Best et al.	**c.1706C>G:p.Pro569Arg**	Missense mutation	PHA	Neutrophil Pelger−Huet phenomenon
			Heterozygous		
#2	Peter Clayton et al. (Fetus B)	**c.1706C>G:p.Pro569Arg**	Nonsense mutation	GRBGD	1. Narrow thorax
			Heterozygous		2. Generalized hydrops fetalis
		**c.1748G>A:p.Arg583Gln**	Missense mutation		3. Ectopic calcification centers
			Heterozygous		4. Intrauterine growth retardation
					5. Long bones were severely shortened and moth-eaten bones
#3	Borovik L et al.	**c.1757G>A:p.Arg586His**	Missense mutation	PHASK	1. Short stature
			Heterozygous		2. Hyperlordosis
		c.651-653delCATins	Nonsense mutation		3. Mild kyphosis
		TGATGAGAAA:p.Ile218Aspfs*19	Heterozygous		4. Short finger-shaped and dumbbell-shaped Pelger−Huet cells
#4	Mchugh D et al.	**c.1747C>T:p.Arg583Term**	Nonsense mutation	PHA	1. Neutrophil nuclei are lobed (bilobed or rod-shaped)
			Heterozygous		2. Rough chromatin
#5	Eliza Thompson et al. (patient 1)	c.1504C>G:p.Arg502Gly	Missense mutation	GRBGD	1. Short stature 2. Dysplasia of the metaphysis of the spine
			Heterozygous		
		**c.1748G>T:p.Arg583Leu**	Missense mutation		
			Heterozygous		

Bold values is exon 14 mutation.

In reference to multiple studies (Sobreira Net al., 2015; M.D.F. Carvalhoet al., 2017; Waterham et al., 2003), the defects in the *LBR* gene were found to be associated with mild skeletal phenotypes, including missense, nonsense, or frameshift variants, and these defects were distributed throughout the nucleoplasmic and transmembrane regions of proteins. In addition, one of the two pathogenic variants involves exons 12, 13, and 14.

A homozygous variant (c.1757G>A(p.Arg586His)) was reported in both fetuses for the first time in our study. Homozygous pathogenic variants were more common in probands born to consanguineous parents due to common ancestors. In the family, we report that the homozygous pathogenic variants came from unrelated parents, which can be explained by recessive consanguinity. In future studies, we can try to explore this mystery. Although, prenatal long bone measurements belong to the fatal skeletal dysplasia in the two fetuses. Based on prenatal USG and X-rays findings, both fetuses appeared to have a moderate skeletal dysplasia, similar to that reported by Thompson *et al* *.*

Although prenatal measurements of long bones are fatal for fetal skeletal dysplasias, according to the results of prenatal USG and X-ray examination, both fetuses appeared to have moderate skeletal dysplasia, which was similar to that reported by Thompson et al.

Standardized and systematic ultrasound examination is the basis for the prenatal detection of skeletal dysplasias. Although prenatal ultrasound examination of a lethal skeletal dysplasia is not a difficult task, there is an overlap in ultrasound imaging findings between different types of lethal and non-lethal skeletal dysplasias. Therefore, on the basis of strict use of standard measurement methods, the use of USG for the prediction and diagnoses of prenatal fatal and non-fatal skeletal dysplasias still have some limitations. According to AlphaFold, we can confirm that Arg586 is at a conserved residue in the 3D folding of 3-β-hydroxysterol δ14-reductase. We can not only find that Arg586 is in the α-helix, it is also found to be linked to ASP450 and GLU208 as a donor of hydrogen bonds (pLDDT score: 88.50).

Nevertheless, we have not scored this mutation through the DeepMind pipeline because there are still several limitations to the current implementation of AlphScore. Indeed, we do align the structure between the mutation and reference. Although there is no significance in the α-helix in Arg586 residues, it has an effect on the next α-helix. The predicted mutation protein structure was constructed using AlphaFold and compared using PyMOL (https://colab.research.google.com/github/ sokrypton/ColabFold/blob/main/AlphaFold2.ipynb#scrollTo=UGUBLzB3C6WN). According to the aforementioned analysis, the effect of Arg586 on the next α-helix may be related to the moderate skeletal dysplasia of both fetuses in this Chinese family.

In conclusion, the c.1757G>A(p.Arg586His) variant located at the end of the transmembrane domain 8 results in a moderate skeletal dysplasia phenotype. With the limitations of ultrasound diagnosis, whole-exome sequencing can help clinically, especially, in genetic counseling to carry out more scientific phenotyping and expanding the phenotypic spectrum of the disease.

## Data Availability

The data presented in the study are deposited in the repository of Sequence Read Archive (SRA), accession number is SUB12035353, https://submit.ncbi.nlm.nih.gov/subs/sra/SUB12035353.
